# Appraisal of the role of external skeletal fixation in the management of sequelae of open tibial fractures

**DOI:** 10.4103/0019-5413.43388

**Published:** 2008

**Authors:** Mahmound A El-Rosasy

**Affiliations:** Department of Orthopaedic Surgery and Traumatology, Faculty of Medicine, University of Tanta, Al-Geish Street, Tanta – Egypt

**Keywords:** Open fracture, tibia, sequelae, nonunion, deformity, external fixation

## Abstract

**Background:**

Severe open tibial fractures are more apt to be followed by complications even with the universally accepted lines of treatment. The present study investigated the role of external skeletal fixation, based on Ilizarov techniques, in the management of the sequelae of open tibial fractures with modifications to meet the requirements of each case.

**Materials and Methods:**

We reviewed the results of treatment of 148 cases of late presentation with complicated open tibial fractures. Their ages ranged from 12 to 74 years (average, 34 years). Active infection was present in 40 cases. We performed acute shortening and relengthening in 60 cases; excision of nonunion, acute deformity correction, and lengthening for nonunion with deformity in 30 cases; segmental excision and bone transport in 20 cases; gradual deformity correction after osteotomy in 15 cases; and distraction and gradual deformity correction for hypertrophic nonunion with deformity in 23 cases. Ilizarov external fixator was used in 96 (65%) cases, and monolateral fixator was used in 52 (35%) cases. The mean follow-up was 35 months (range 24 to 118 months).

**Results:**

Fracture union was achieved in all cases (100%). Evaluation of results were based on both objective (clinical and radiological) and subjective criteria and patients' satisfaction. The results were satisfactory in 139 cases (94%) and unsatisfactory in nine (6%) cases because of residual leg length discrepancy, joint stiffness, and persistent pain.

**Conclusions:**

The use of external fixation, based on Ilizarov techniques, is invaluable in the management of difficult open tibia fractures. However, the technique should be tailored to the requirements of each case. The functional outcome is predetermined by the soft tissue status before treatment.

## INTRODUCTION

Open tibial fractures are more apt to be followed by complications even with the universally accepted lines of treatment.^1^ Despite the progress in the field of orthopedic trauma, problem cases are often encountered. The basic cause is the severity of injury, the method of treatment at the first place, and the patient's general condition prior to injury. Complications of open tibial fracture often develop because of infection or inappropriate mechanical or biological environment. The usually confronted complications are combinations of infection, nonunion, malunion, and bone and soft tissue loss.^1^–^4^ Several treatment protocols using soft tissue and bone flaps in a staged reconstruction have been described. The functional outcome of the traditional treatment methods may be unsatisfactory because of residual deformity and leg length discrepancy (LLD).^5^–^8^ The use of external fixation, using the tension stress effect (Ilizarov principle), would help salvage limbs that were previously considered due for amputation.^9^–^11^ The Ilizarov techniques have problem of difficult application as well as problem of patients' acceptance. The bone transport and docking site problems are technique related.^12^–^14^ The present study investigated the role of external skeletal fixation, based on Ilizarov techniques, in the management of the sequelae of open tibial fractures with modifications to meet the requirements of each case.

## MATERIALS AND METHODS

This retrospective study included 148 patients who were treated for the sequelae of open tibial fractures between 1997 and 2005.

**Figure 1:**
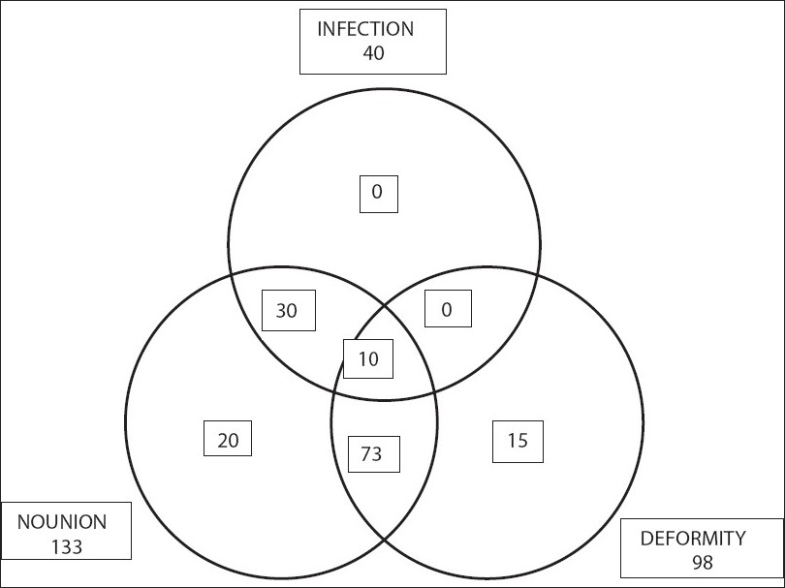
The combinations of complications in the study cases

The inclusion criterion was that the patient should have one or more complications secondary to the open tibial fracture either because of the injury itself or as a result of previous surgical interference. The combinations of the complications are shown in figure 1. Patients who were not candidates for limb reconstruction using external skeletal fixation due to intolerance to the technique and cases that could be managed by simpler techniques were excluded from the study. The patients' ages ranged from 12 to 74 years (average, 34 years). The time elapsed between the injury and our treatment ranged from one week to 160 weeks (average, 33 weeks). Comorbidities were present in 32 cases (22%) in the form of Type II diabetes mellitus, cigarette smoking, and morbid obesity [Table 1].

**Table 1:** Preoperative difficulty indicators

Preoperative difficulties	N	%
Comorbidities (diabetes mellitus, smoking, obesity)	32 cases	22
Leg length discrepancy	1-11 cm	Average 4.3 cm
Deformity (oblique plane)	10-35°	Average 27°
Ankle joint stiffness	12 cases	8
Level of fracture		
Upper third	28 cases	19
Middle third	84 cases	57
Lower third	36 cases	24
Active infection	40 cases	27
Bone loss	1-13 cm	Average 5.2 cm
Equines contracture (>5°)	29 cases	20

### Preoperative evaluation

Preoperative counseling of the patient and his caregivers was carried out with the aim of explaining the intended procedure and the expected difficulties to ensure the patient's cooperation and the support of his family. The smoker patients were requested to quit smoking before embarking on the procedure.

General clinical examination of the patient was done to exclude the presence of general debilitating or systemic illness. The limb was examined for the neurovascular status, skin condition, stiffness of the nonunion, shortening, and range of motion at adjacent joints. Radiographs of the limb were obtained and examined for the type and level of the nonunion, bone quality, size of bone defect, deformity analysis, bone shortening, and the condition of nearby joints.^15^

### Technique selection

Based on the data collected from the preoperative evaluation, the methods of interference were planned: (a) acute shortening and relengthening (ASRL) were performed when dealing with atrophic nonunion with bone loss [Figure 2];^16^ (b) segmental excision and bone transport was preferred if ASRL was deemed unsafe due to fibrotic skin secondary to a long-standing infection or multiple longitudinal scars [Figure 3];^11^,^16^ (c) distraction of hypertrophic nonunion with gradual deformity correction was performed [Figure 4]^17^ and (d) corrective osteotomy and gradual deformity correction was used for malunited fractures not amenable to acute deformity correction and internal fixation. A preconstructed Ilizarov fixator was applied to the leg matching the deformity, and then a percutaneous drill-holes osteotomy was performed at the center of rotation of angulation (CORA).^15^,^18^ Gradual distraction of the osteotomy was done to correct the deformity and equalize the legs' length.

**Figure 2A:**
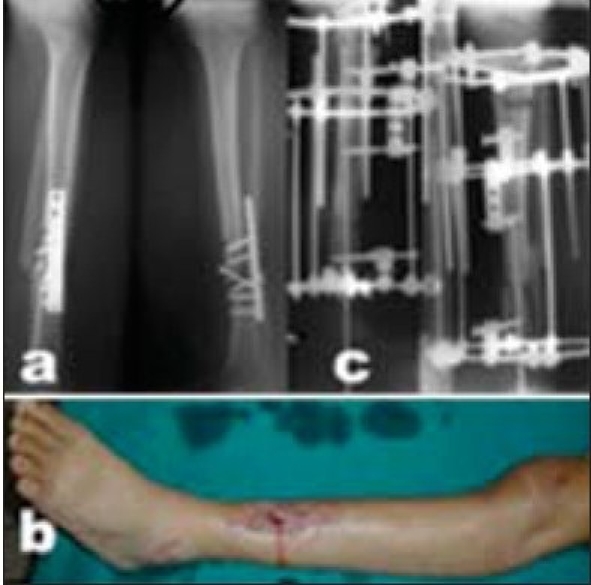
(a) Radiograph (Lateral and anteroposterior) of 23-year-old male patient, who was treated by plate and screws fixation for Type II open tibia fracture, shows implant failure due to infection and atrophic nonunion. (b) Clinical photograph 8 months after trauma with infected nonunion. (c) Postoperative radiograph (Lateral and anteroposterior) after hardware removal, excision of the nonunion and infected tissues, acute limb shortening (docking) over Rush rod and application of Ilizarov fixator for compression-distraction.

**Figure 2B:**
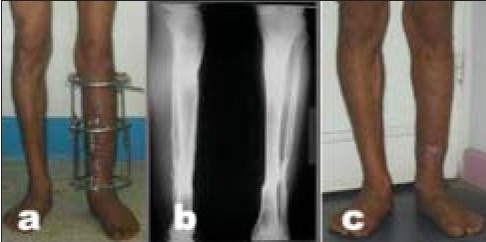
(a) Clinical photograph during treatment, the patient was allowed weight-bearing. (b) Follow-up radiographs (lateral and anteroposterior) showing consolidation of the nonunion and limb lengthening of 7 cm. (c) Clinical photograph showing equal limb length.

**Figure 3A:**
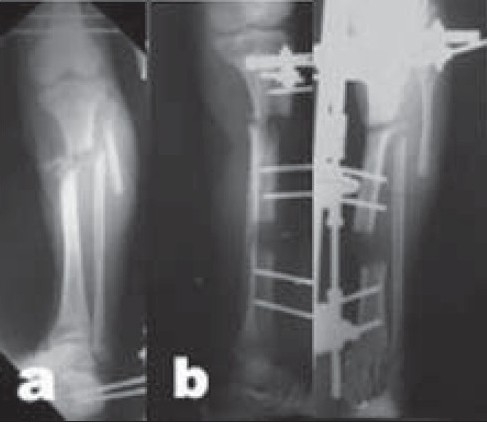
26-year-old male patient who sustained Type IIIB open fracture of the tibia. (a) Radiograph of the leg (anteroposterior view) after being treated elsewhere by gastrocnemius muscle flap, antibiotic beads insertion, shows spanning external fixator. (b) The patient was then referred after application of a monolateral fixator for bone transport. Radiograph of the same leg (lateral and anteroposterior view) shows deviation of the transported segment and distraction at the nonunion site.

**Figure 3B:**
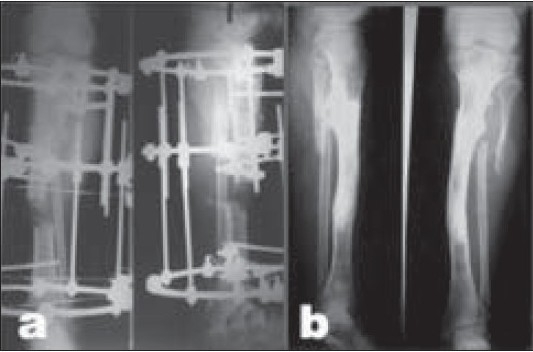
(a) Radiograph of the same leg (lateral and anteroposterior view) with Ilizarov fixator after conversion from monolateral fixator with insertion of bone graft. (b) Followup radiograph (lateral and anteroposterior view) shows consolidation of the nonunion and limb lengthening of 6 cm.

**Figure 4A:**
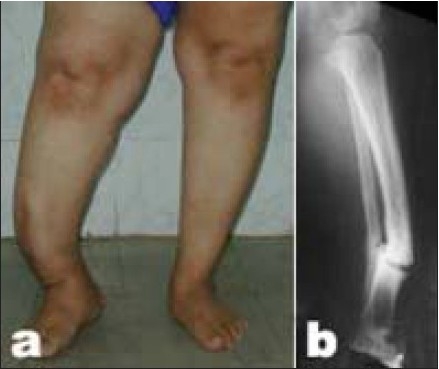
(a) Clinical photograph and (b) Radiograph (lateral view) of a female patient aged 51 years with leg deformity after being treated with interlocking nail for Type II open distal tibial fracture which shows hypertrophic nonunion after nail extraction following infection.

**Figure 4B:**
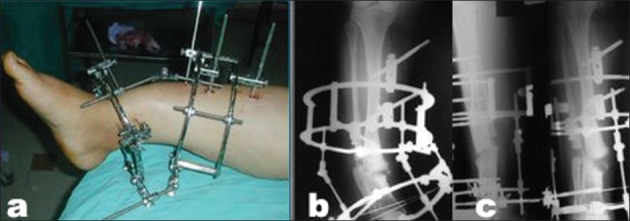
(a) Intraoperative clinical photograph shows the application of a preconstructed Ilizarov fixator and fibular osteotomy for closed distraction of the nonunion. (b, c) Postoperative radiographs show gradual deformity-correction.

**Figure 4C:**
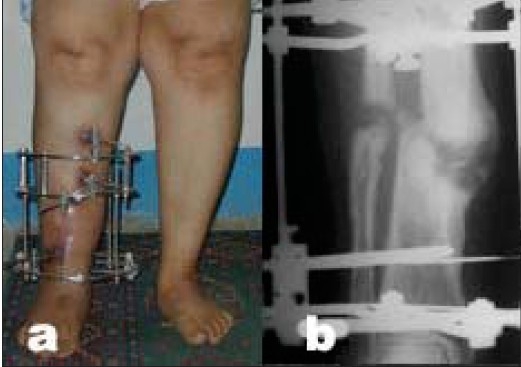
(a,b) Clinical photograph and radiograph (anteroposterior view) result after complete deformity correction in consolidation phase of the distraction gap while weight bearing is allowed.

The choice of the method of fixation was governed by the preoperative difficulty indicators [Table 1]. Ilizarov external fixator was used for limb reconstruction when the required limb lengthening exceeded 5 cm with osteoporotic bones. The patient needed gradual deformity correction and multilevel surgery with necessary inclusion of the foot in the fixator. Monolateral fixator was used for linear distraction-compression in patient with good bone quality, and short segment lengthening. Hybrid fixation (fixator augmented be intramedullary rod) was needed when dealing with short distal bone segments, acute shortening of more than 5 cm, severe osteoporosis, and stiff ankle joint.^16^

### Postoperative management

Postoperative leg elevation was necessary in cases of acute limb shortening to avoid postoperative edema, which would cause vascular compromise and late ischaemia. Active non–weight-bearing exercises of the limb were encouraged as soon as postoperative pain had settled. Weight-bearing as tolerated was allowed throughout the treatment. Patients were seen in the clinic every two weeks during the distraction phase and then every month during the consolidation phase. Radiographs were obtained in each visit to check the progress of deformity correction, lengthening, and bone healing. The external fixator was removed after radiological bone consolidation (at least three intact bone cortices) and clinical bone union (patient was able to bear weight unsupported with no pain). After fixator removal, a well-fitted plaster cast was applied for one month with weight-bearing. Patients were then followed up every three months and then yearly.

### Evaluation of treatment outcome

The results were assessed based on both objective (clinical and radiographic evaluation) and subjective criteria (limb function and patient's satisfaction) using our system of results evaluation [Table 2].^16^ This included the evaluation of bony union, residual deformity, residual leg length discrepancy, recurrent infection, soft-tissue healing, permanent joint contracture, persistent pain, return to previous work, and patient satisfaction. The final results were considered to be satisfactory or unsatisfactory based on these findings using the summation of these values in all cases. To be considered as satisfactory, the case should fulfill all the criteria of satisfactory results.

**Table 2:** Evaluation of the results^16^

Parameter	Satisfactory	Unsatisfactory
Bony union	United	Not united
Residual deformity	<5°	>5°
Residual leg length discrepancy	<2.5 cm	>2.5 cm
Recurrent infection	No more infection	Bone and/or soft-tissue infection
Soft tissue healing	No exposed bone	Soft-tissue defect remaining
Permanent joint contracture	<5°	<5°
Persistent pain	No or mild pain	Moderate or incapacitating pain
Return to previous work	Yes	Has to change job
Patient's satisfaction	Satisfied	Not satisfied

## RESULTS

In this study, 148 patients were treated for sequelae of open tibial fractures. Their ages ranged from 12 to 74 years (average, 34 years); in the age group between 20 and 50 years, there were 107 cases (72%).

There was previous surgical treatment using different techniques for bone and soft tissue reconstruction in 121 cases (82%). The number of procedures per case ranged from one to five (average, 2.1 procedures/case). The remaining 27 patients (18%) did not have any reconstructive procedures, except for primary debridement and plaster cast immobilization performed in local hospitals.

The method of fixation varied according to the nature of each case; only Ilizarov fixator was used in 71 cases (48%), Ilizarov fixator augmented by intramedullary rod was used in 25 cases (17%), and monolateral fixator was used in 52 cases (35%). Autogenous iliac crest bone graft (ICBG) was used in 97 cases (65%) of atrophic nonunion, and as a secondary procedure in four cases of refracture at the original nonunion and in one case of delayed union following corrective osteotomy.

Four cases (3%) had refracture at the original nonunion, three of whom were in the group of bone transport and one in the ASRL group. All united after another monolateral fixator and ICBG application and remained so until the time of last follow-up. Peroneal nerve palsy developed in one case of distraction of hypertrophic nonunion and gradual deformity correction. Exploration of the peroneal nerve revealed stretching of the nerve on the proximal tibiofibular wire, which was removed and replaced by a half wire from the medial aspect. The nerve palsy resolved with no residual deficit. Frequent superficial pin tract infection occurred in 87 cases (59%) and was treated by daily pin site care and oral antibiotics. No cases of deep infection were encountered during treatment or at follow-up.

Equinus contracture of more than 10° developed in six cases (4%) and was treated by frame extension on the foot and gradual stretching in cases of Ilizarov fixator application (two cases) and by percutaneous achilles tendon lengthening and foot splint application in cases of monolateral fixator application. Limb lengthening was done in all cases and ranged from 1 to 10 cm. Equalization of limb length was obtained in 117 cases (79%), residual LLD less than 2.5 cm occurred in 22 cases (15%), and residual LLD more than 2.5 cm occurred in nine cases (6%). The main reasons for residual LLD were cessation of limb lengthening either due to the patient's intolerance to the procedure or development of significant joint contracture.

Fracture union was obtained in all cases (100%). No cases have developed vascular injury or ischemia due to treatment. Amputation was not needed in any case. The mean follow-up was 35 months (range, 24 to 118 months). The results were satisfactory in 139 cases (94%) and unsatisfactory in nine (6%) cases due to residual leg length discrepancy, joint stiffness, and persistent pain.

## DISCUSSION

The management of open tibial fractures remains a controversial subject because of the lack of clear guidelines regarding the most suitable treatment for each injury pattern. Sequelae of these severe injuries are usually the result of poor management at the first place where the patient was received late or delayed referral to specialized centers.

The combination of the problems of nonunion, deformity, infection, bone loss, and LLD renders traditional treatment protocols fraught with complications due to their invasive nature. The use of open surgical procedures and internal fixation is more liable to end with unsatisfactory functional results, which might prompt the patient and the surgeon to embark on amputation rather than limb reconstruction.

The introduction of limb reconstruction techniques, based on the tension stress effect, has provided the surgeons with a powerful armamentarium for limb salvage.^4^,^9^,^10^

The widespread use of external skeletal fixation based on Ilizarov techniques has revealed several difficulties pertinent to the technique due to patient's inconvenience with the device. On the other hand, the process of bone transport and docking of bone ends could be hindered by fibrous tissue obstruction, skin invagination, segment deviation, and premature consolidation of the osteotomy.^12^–^14^

As a prerequisite for treatment, it is essential to recognize and define the existing complications of the open tibial fractures, and consequently the management would be successfully planned. The interference should be planned based on the requirements of the case to avoid over- or undertreatment. We have defined the difficulty indicators that would influence the choice of the method of fixation, where the choice shifts toward the use of Ilizarov fixator as the local risk factors increase. However, such complex management would not be suitable in the presence of certain factors related to the patient's health, e.g., morbid obesity and chronic illness [Table 1].

The technique of ASRL has been proved to be effective in converting a complex limb reconstruction into a simpler procedure and obviates the problems related with bone transport.^13^,^14^,^16^ However, it should be avoided in the presence of soft tissue fibrosis or multiple longitudinal scars to avoid acute ischemia. Acute correction of deformities, after nonunion excision and acute shortening, allows the application of monolateral external fixator for linear distraction-compression. The use of monolateral fixator is much simpler to apply than ring fixators and better tolerated by the patient.^13^,^16^

In cases of hypertrophic nonunion with deformity, the procedure is performed percutaneously, and the Ilizarov fixator is preferred because it is more versatile for gradual deformity correction. The fixator application can be modified, to have a reduced size using mostly half pins and fewer rings, for easier application [Figure 4]. Limb lengthening can be performed at the same time through the distraction of the nonunion and deformity correction.^17^

The correction of malunited fractures of large magnitude is ideally performed through the CORA (osteotomy rule I); otherwise, the osteotomy should be placed at the nearest location with good bone quality and soft tissue coverage (osteotomy rules II and III).^15^

Cigarette smoking has been shown to adversely affect bone healing, and the patient should quit smoking before embarking on this procedure.^19^ In their study, Marsh *et al*, reported that heavy smoking appeared to have deleterious effect on the maturation of the newly formed callus by distraction.^1^ McKee *et al*, demonstrated in their study that smoking is associated with the development of nonunion when using Ilizarov technique for the reconstruction of the femur and tibia.^20^ On the other hand, any general debilitating disease condition should be corrected to optimize the environment for this complex procedure.

The use of external skeletal fixation has been effective in the management of complicated limb reconstructions with predictably good bone results. However, this does not guarantee equally good functional outcome, which is predetermined by the soft tissue status before treatment.^11^ The surgeon's experience and patient's commitment are other contributory factors that influence the treatment results.

## CONCLUSION

The use of external fixation, based on Ilizarov techniques, is invaluable in the management of the sequelae of open tibial fractures; however, the technique should be tailored to the requirements of each case.
